# Apalutamide-induced drug reaction with eosinophilia and systemic symptoms and Stevens-Johnson syndrome overlap successfully treated with cyclosporine and benralizumab: A case report and literature review

**DOI:** 10.1016/j.jdcr.2026.08.045

**Published:** 2026-09-03

**Authors:** Alaa Erras, Zachary J. Jaeger, Lena Chu, Helen H. Park, Brian R. Hinds, Reid Oldenburg

**Affiliations:** Department of Dermatology, University of California San Diego, School of Medicine, La Jolla, California

**Keywords:** androgen receptor antagonist, apalutamide, ARSI, benralizumab, bulla, CCL1, CCL17, CCR4, CCR8, corticosteroids, CsA, CXCL10, CXCR3, cyclosporin A, cyclosporine, darolutamide, dermal dendritic cells, dermatitis, desquamation, DIHS, DRESS, DRESS-SJS overlap, drug reaction with eosinophilia and systemic symptoms, drug-induced hypersensitivity syndrome, enzalutamide, eosinophilia, eosinophils, erythroderma, exanthem, IL-5 inhibitor, immunosuppression, interface, interleukin-5, JAK inhibitor, J-SCAR, keratinocytes, prostate cancer, rash, RegiSCAR, severe cutaneous adverse reaction, SJS, SJS/TEN, Stevens-Johnson syndrome, TARC, TEN, Th2, toxic epidermal necrolysis

## Introduction

Drug reaction with eosinophilia and systemic symptoms (DRESS) is a life-threatening medication-induced hypersensitivity reaction characterized by delayed onset of morbilliform rash, facial edema, eosinophilia, and multi-organ involvement.^1^^,^^2^ Stevens-Johnson syndrome (SJS) exhibits full-thickness epidermal necrosis on histopathology. DRESS is clinically distinguished from SJS by longer latency, end-organ damage, eosinophilia, and human herpesvirus reactivation.

Apalutamide, an androgen receptor signaling inhibitor (ARSI) used to treat prostate cancer, has been associated with increased risk of severe cutaneous adverse reactions (SCAR).^3^ We report a challenging case of DRESS with SJS-like phenotypic overlap presenting 9 days after apalutamide initiation, refractory to high-dose corticosteroids and successfully treated with cyclosporine and benralizumab.

## Case report

A 68-year-old Filipino man with locally advanced prostate cancer (pT3bN1, Gleason 4 + 5) underwent radical prostatectomy in 2017, followed by adjuvant radiation and androgen deprivation therapy. He developed biochemical recurrence in 2023, prompting reinitiation of androgen deprivation therapy and subsequent addition of apalutamide for castration-resistant disease. Nine days after starting apalutamide, he developed a painful, pruritic, erythrodermic eruption with facial edema and generalized bullae without ocular involvement (Fig 1), accompanied by fever, fatigue, dysphagia, and dyspnea. Work-up revealed hypereosinophilia (peak absolute eosinophil count 7200/μL), hepatitis (alanine aminotransferase 373 U/L), and acute kidney injury (creatinine 2.1 mg/dL, blood urea nitrogen 37 mg/dL). A RegiSCAR score of 6 supported the diagnosis of DRESS.^1^^,^^4^

**Fig 1:**
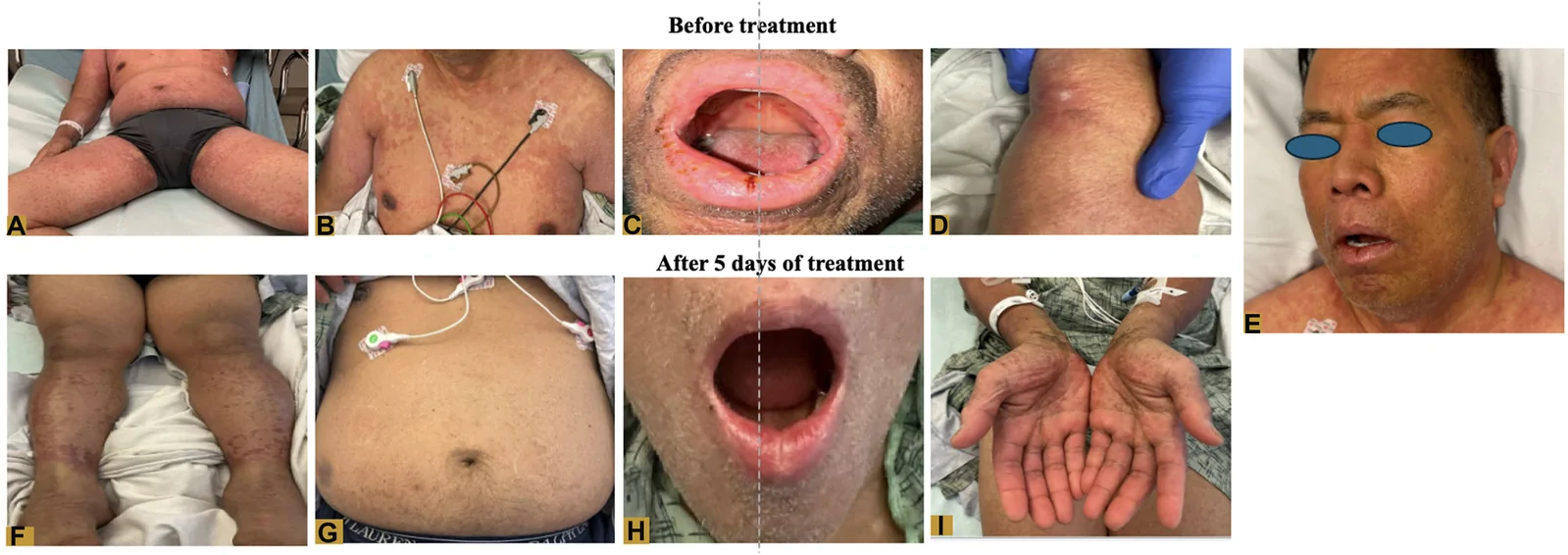
DRESS with SJS before and after treatment. **A** and **B,** Diffuse erythematous coalescing macules and papules on trunk and bilateral upper and lower extremities before treatment. **C,** Shallow erosions to upper and lower lips and hard palate before treatment. **D,** Tense bulla with erythematous and edematous base on right lateral wrist before treatment. **E,** Diffuse erythema of the face sparing the periorbital region. **G,** Interval improvement of macular and patchy erythema on trunk and **(F)** dusky erythema and violaceous papules on bilateral lower extremities after 5 days of treatment. **H,** Interval improvement of erythema of buccal mucosa, no erosions noted after 5 days of treatment. **I,** Erythematous macules on palms and wrists. *DRESS*, Drug reaction with eosinophilia and systemic symptoms; *SJS*, Stevens-Johnson syndrome.

Extended laboratory evaluation revealed C-reactive protein 8.34 mg/dL, lactate 3.1 mmol/L, ferritin 2455 ng/mL, interleukin (IL) 5111 pg/mL (normal: ≤2.1), soluble IL-2 receptor/cluster of differentiation 25 5815 pg/mL (normal: 175.3-858.2), tumor necrosis factor-alpha 2.8 pg/mL (normal: ≤7.2), and chemokine(C-X-C) ligand 9 >2000 pg/mL (normal: <179). Complete human herpesvirus studies and *Strongyloides* IgG were unremarkable. Hemophagocytic lymphohistiocytosis (HLH) was excluded by H-score (87, <1% probability).^5^

Histopathology demonstrated intense interface dermatitis with apoptotic keratinocytes extending into adnexae along with tissue eosinophilia, consistent with SCAR.^6^^,^^7^ Marked papillary dermal edema resulted in focal detachment of the partially necrotic epidermis, correlating with bullae and desquamation suggestive of SJS-phenotypic overlap.

Apalutamide was promptly discontinued, and intravenous methylprednisolone 125 mg daily (1.5 mg/kg/day) was initiated. After 3 days, there was little improvement in bullous erythroderma, and laboratory analysis demonstrated ongoing end-organ damage despite high-dose systemic corticosteroids. Cyclosporine was initiated at 200 mg twice daily (4.8 mg/kg/day), and within 24 hours, the patient reported ∼30% improvement in the rash, along with 65% reduction in eosinophilia (Fig 2). Cyclosporine was held after 2 doses due to uptrending transaminases. Due to refractory eosinophilia and elevated IL-5 levels, benralizumab 30 mg was administered subcutaneously, with rapid normalization of eosinophilia. Liver enzymes also improved, and cyclosporine was restarted without further elevations.

**Fig 2:**
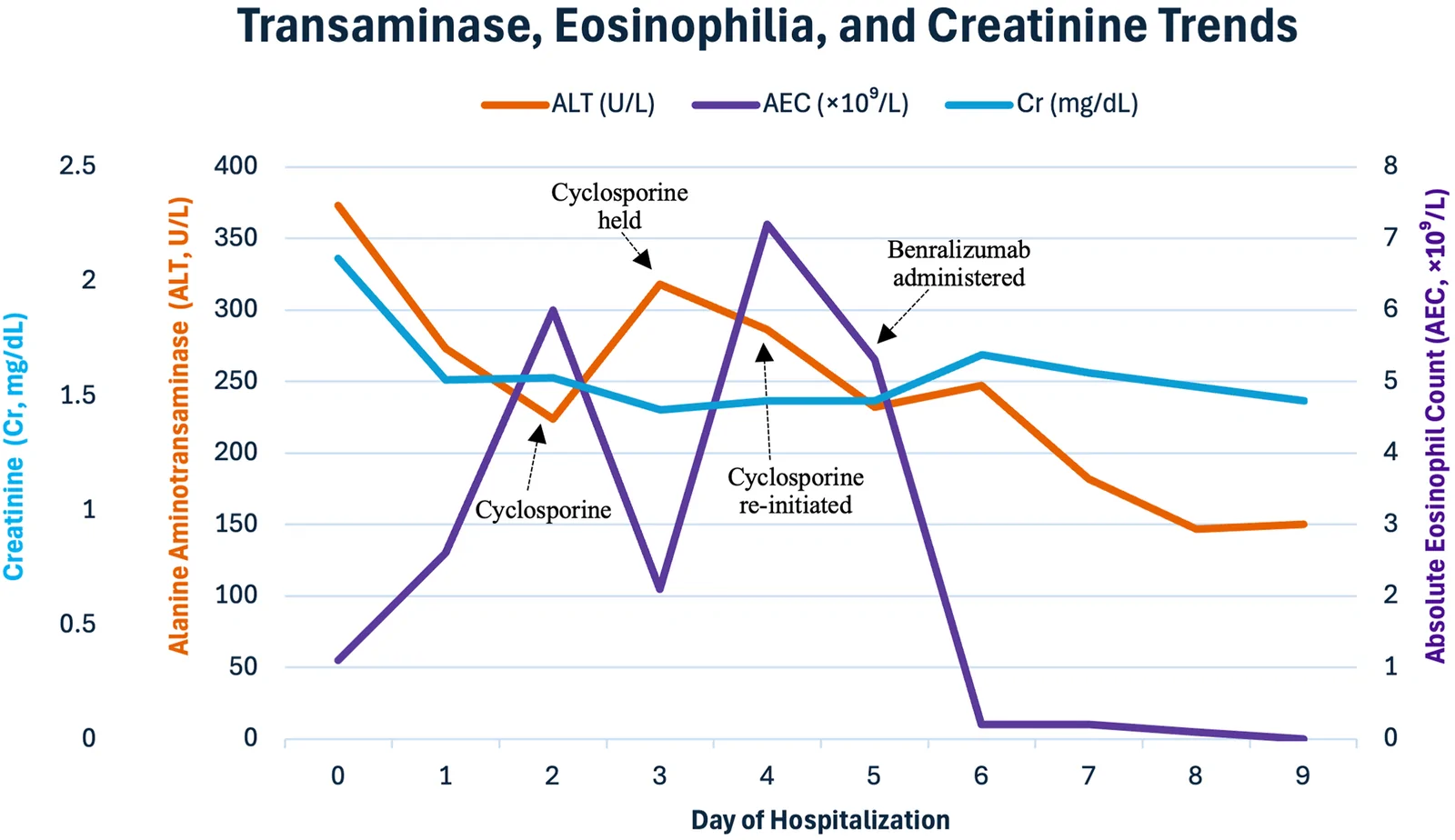
Transaminase, eosinophilia, and creatinine trends during hospitalization with annotations marking key treatment interventions. *AEC*, Absolute eosinophil count; *ALT*, alanine aminotransferase; *Cr*, creatinine. This figure is the authors’ original work.

As the rash resolved, the patient was discharged on oral prednisone and cyclosporine tapers. Cyclosporine was continued for 11 days after discharge then tapered to 100 mg twice daily for 10 days before discontinuation. Methylprednisolone was reduced to 100 mg daily during admission and converted to prednisone 80 mg daily upon discharge, decreasing by 10 mg every 5 days. The patient achieved full recovery without long-term sequelae after 10-m outpatient follow-up.

## Discussion

DRESS pathophysiology involves complex immunological mechanisms mediated by multiple T-cell populations.^8^ A prominent Th2 response distinguishes DRESS from other SCARs; keratinocytes and dermal dendritic cells produce thymus and activation-regulated chemokine, recruiting Th2 cells to the skin via chemokine receptor type 4.^2^^,^^9^ Th2 lymphocytes release IL-5, driving eosinophil recruitment, activation, and migration into tissues.^8^^,^^10^ Activated eosinophils release cytotoxic granule proteins, including eosinophil cationic protein, that may contribute to tissue injury and organ involvement.^2^^,^^9^^,^^11^, ^12^, ^13^ Concurrently, cytotoxic CD8+ T cells and Th1 responses further amplify inflammatory injury through tumor necrosis factor-alpha, interferon-gamma, and cytotoxic mediators.^2^ Proteomic analysis has revealed distinct immune signatures involving type 1, type 2/eosinophilic, and type 3/innate immunity endotypes, with disease severity correlating significantly between CXCL9/10 and interferon-gamma levels.^14^ In recalcitrant disease, memory T-cell activation via the chemokine receptor type 8/chemokine (C-C motif) ligand 1 and chemokine receptor type 3/chemokine (C-X-C motif) ligand 10 axes corresponds to in vitro responsiveness to JAK inhibition.^15^ Serum IL-5, absolute eosinophil count, and C-reactive protein levels correlate with disease activity and severity and are useful for clinical monitoring.^8^

Apalutamide-induced SCARs are increasingly reported, most notably DRESS and SJS/ toxic epidermal necrolysis (Table I). In the TITAN trial, 27.1% of apalutamide-treated patients reported any rash, 6.3% grade 3 or higher.^16^^,^^17^ The incidence of cutaneous reactions among Japanese populations reaches 50%, with 14.7% being grade 3.^17^, ^18^, ^19^ In the Food and Drug Administration Adverse Event Reporting System, apalutamide accounted for the majority of ARSI-associated SCAR reports (*n* = 77), compared to enzalutamide (*n* = 25) and darolutamide (*n* = 1), with a disproportionate number coming from Japan (*n* = 24).^3^ Mechanistic studies have suggested that apalutamide’s 2-cyanopyridine moiety may bind cysteine residues to form hapten conjugates that trigger adverse immune responses; this moiety is replaced by a 2-cyanophenyl group in enzalutamide, possibly accounting for its lower SCAR incidence.^3^ Our literature review suggests considering an alternative ARSI in Asian patients to reduce risk of SCAR.

**Table I:** Summary of reported cases of apalutamide-induced DRESS and SJS/TEN

Authors (y)	Age/sex	Time to onset (d)	Type of reaction	Main clinical features	Histopathology	Laboratory findings	Treatment	Outcome
Akai et al (2025)^20^	78/M	35	DRESS	RegiSCAR not reported; fever; violaceous facial and truncal erythema with purpura of the lower limbs	Dyskeratotic cells in the epidermis with papillary edema and a perivascular lymphocytic infiltrate with eosinophils in the upper dermis	Eosinophilia; leukocytosis; elevated transaminase; elevated LDH	Oral prednisolone 60 mg/d	Recovery
Gil-Lianes et al (2024)^21^	76/M	108	DRESS	RegiSCAR 2; maculopapular rash without mucosal involvement; exfoliative erythroderma	Subacute spongiotic dermatitis with lymphocytic exocytosis and a mixed papillary dermal infiltrate of lymphocytes, neutrophils, and eosinophils	Eosinophilia; leukocytosis	Systemic corticosteroids; oral prednisone; oral antihistamine (dose and taper not reported)	Recovery
Hallamies et al (2024)^22^	70/M	90	DRESS	RegiSCAR not reported; fever; scaly erythroderma; LAD	Spongiotic epidermis and a dermal infiltrate of lymphocytes and numerous eosinophils	Eosinophilia; monocytosis	IV corticosteroids (agent and dose not reported)	Not reported
	72/M	Not reported	DRESS	Grade 3/NCI-CTCAE 3 maculopapular exanthema without mucosal involvement; fever; LAD	Interface dermatitis with eosinophils	Eosinophilia; elevated transaminases	Oral prednisolone 40 mg/d, tapered over 1 mo	Not reported
Wang et al (2023)^23^	77/M	40	SJS	Naranjo 6; crimson maculopapular rash with buccal mucosal ulcers; fever; LAD	Interface dermatitis with confluent epidermal necrosis and subepidermal split with superficial perivascular infiltration of lymphocytes	Not reported	IV methylprednisolone 60 mg/d; human serum albumin; TNF-α inhibitor	Recovery
	69/M	122	SJS	Naranjo 6; florid papules and plaque on dorsum of feet	Epidermal necrosis and perivascular infiltrate of lymphocytes and eosinophils	Not reported	IV methylprednisolone 40 mg/d; oral thalidomide 25 mg/d; topical ointments	Recovery
	72/M	45	SJS	Naranjo 6; maculopapular erythema with mucosal involvement.	Not reported	Not reported	IV methylprednisolone 40 mg/d	Recovery
	74/M	Not reported	TEN	Naranjo 9; SCORTEN 5; necrosis of buccal and urethral mucosa with exudation	Not reported	Not reported	IV methylprednisolone 80 mg/d; single dose of adalimumab 80 mg	Death due to multi-organ failure within 3 d
Flynn et al (2023)^24^	83/M	∼35	TEN	SCORTEN 3; widespread dusky erythematous maculopapular eruption with epidermal detachment, bullae, and positive Nikolsky sign	Prominent interface dermatitis with a perivascular dermal infiltrate of lymphocytes and scattered eosinophils and neutrophils	Eosinophilia; mild normocytic anemia; elevated CRP	Initially refractory to IV methylprednisolone 2 mg/kg/d × 5 d and IVIG 2 g/kg × 2 d; adjunct broad-spectrum IV antibiotics and topical emollients; transitioned to oral corticosteroids	Death (unrelated to SCAR; fall with hip fracture 4 wk post-discharge)
Martin et al (2023)^25^	74/M	14	DRESS	RegiSCAR 5; diffuse erythematous maculopapular rash with LAD	Spongiotic dermatitis with a dermal polymorphous infiltrate with eosinophils	Eosinophilia; leukocytosis	Oral prednisone 10 mg TID; famotidine 20 mg BID; levocetirizine 5 mg BID	Recovery
Ducharme et al (2022)^26^	85/M	39	DRESS	RegiSCAR 7; confluent erythematous plaques and facial edema without mucosal involvement	Interface dermatitis with a dermal infiltrate of eosinophils	Eosinophilia; elevated transaminases	IV corticosteroids 0.5 mg/kg per d (agent not specified)	Recovery
Hsu et al (2022)^17^	85/M	30	DRESS with features of SJS overlap	RegiSCAR not reported; generalized erythematous maculopapular rash with fever, facial edema, and periorbital sparing; subsequent painful erythroderma and large flaccid blisters with positive Nikolsky sign	Severe interface dermatitis with complete subepidermal split; perivascular lymphocyte infiltration with abundant eosinophils and neutrophils	Eosinophilia; elevated transaminase; elevated pancreatic enzymes	IV methylprednisolone (dose and taper not reported)	Recovery
	77/M	38	DRESS with features of SJS overlap	RegiSCAR not reported; generalized erythema superimposed on jaundiced skin with flaccid bullae, denuded erosions, positive Nikolsky sign, and mucosal erosion	Severe interface dermatitis with full thickness epidermal necrosis and subepidermal split; perivascular lymphocyte and abundant eosinophil/neutrophil infiltration	Eosinophilia; elevated transaminases; hyperbilirubinemia; AKI	Not reported	Not reported
Sagawa et al (2020)^27^	77/M	14	TEN	SCORTEN 6; fever; erythema with atypical target lesions on trunk; skin detachment >30% BSA; hemorrhagic erosions of lips, oral mucosa, and genital region; positive Nikolsky sign; severe dry eyes	Full-thickness epidermal necrosis; mononuclear cell infiltrate in upper dermis	No leukocytosis or eosinophilia	IV methylprednisolone pulse 1000 mg/d × 3 d; IVIG 400 mg/kg/d × 5 d	Death (due to multiorgan failure and rapid metastases on day 53 from rash onset)

Severity and causality assessments are reported as provided in the original publications, including RegiSCAR, SCORTEN, and Naranjo scores. Laboratory abnormalities reflect values reported at presentation or during hospitalization. When treatment dose, taper, histopathology, laboratory findings, or outcomes were not explicitly stated, they are indicated as not reported. Outcomes categorized as recovery indicate clinical resolution of cutaneous and systemic manifestations. Deaths were attributed to complications as described by the reporting authors.

*AKI*, Acute kidney injury; *BID,* twice daily; *BSA,* body surface area; *CRP,* C-reactive protein; *DRESS,* drug reaction with eosinophilia and systemic symptoms; *IV,* intravenous; *IVIG,* intravenous immunoglobulin; *LAD,* lymphadenopathy; *LDH,* lactate dehydrogenase; *Naranjo*, Naranjo Adverse Drug Reaction Probability Scale; *NCI-CTCAE,* National Cancer Institute Common Terminology Criteria for Adverse Events; *RegiSCAR,* Registry of Severe Cutaneous Adverse Reactions; *SCAR,* severe cutaneous adverse reaction; *SCORTEN,* Severity-of-Illness Score for Toxic Epidermal Necrolysis; *SJS*, Stevens-Johnson syndrome; *TEN*, toxic epidermal necrolysis; *TID*, three times daily; *TNF-α*, tumor necrosis factor-alpha.

Among 7 other published cases, median DRESS onset was 38 days (range 14-108) after apalutamide initiation (Table I). While 2 cases of DRESS overlapping with SJS occurred at 30 and 38 days, our case presented with an unusually short latency of 9 days. Higher-grade cutaneous reactions associated with apalutamide appear to correlate with shorter latency.^24^ Potential mechanisms for this shortened latency include an unidentified HLA risk allele enabling more efficient drug presentation or cancer-associated immune dysregulation with impaired regulatory T-cell function. Notably, all 3 overlapping DRESS-SJS cases occurred in patients of Asian descent.^17^ Among published cases, none described apalutamide-induced DRESS refractory to systemic corticosteroids.^2^^,^^13^

We highlight a rare and challenging steroid-refractory case successfully treated with cyclosporine and adjunctive benralizumab. In our patient, marked eosinophilia persisted despite systemic corticosteroids, and cyclosporine was held due to transaminase elevation, necessitating an alternative approach to control eosinophil-driven organ involvement. Benralizumab, an anti-IL-5 receptor alpha monoclonal antibody, prevents eosinophil activation and induces antibody-dependent cell-mediated cytotoxicity, achieving near-complete eosinophil depletion.^2^ Given the central role of Th2-driven IL-5 elevation in DRESS pathogenesis,^2^ benralizumab’s dual mechanism likely reduced eosinophil-mediated tissue injury, supporting the clinical response observed. Cyclosporine^28^ and IL-5-inhibitors such as benralizumab^13^^,^^29^ show promise as personalized therapies for steroid-refractory DRESS in the appropriate clinical setting.

**Key teaching points**

- Apalutamide may confer greater SCAR risk than other androgen receptor signaling inhibitors, particularly in patients of Asian descent.
- Benralizumab may mitigate eosinophil-mediated organ damage in steroid-refractory drug reaction with eosinophilia and systemic symptoms.

## Conflicts of interest

None disclosed.

## References

[1] Calle A.M., Aguirre N., Ardila J.C., Cardona Villa R. (2023). DRESS syndrome: a literature review and treatment algorithm. World Allergy Organ J.

[2] Kroshinsky D., Cardones A.R.G., Blumenthal K.G. (2024). Drug reaction with eosinophilia and systemic symptoms. N Engl J Med.

[3] Liu J., Liu X., Zeng H., Tong Y., Chen Z., Dong Z. (2025). Severe cutaneous adverse reactions associated with second-generation androgen receptor antagonists in prostate cancer patients. PLoS One.

[4] Kardaun S.H., Sekula P., Valeyrie-Allanore L. (2013). Drug reaction with eosinophilia and systemic symptoms (DRESS): an original multisystem adverse drug reaction. Results from the prospective RegiSCAR study. Br J Dermatol.

[5] Fardet L., Galicier L., Lambotte O. (2014). Development and validation of the HScore, a score for the diagnosis of reactive hemophagocytic syndrome. Arthritis Rheumatol.

[6] Ehsan E., Wei E.X., Georgesen C. (2024). Histopathologic features of drug reaction with eosinophilia and systemic symptoms. Am J Dermatopathol.

[7] Ortonne N., Valeyrie-Allanore L., Bastuji-Garin S. (2015). Histopathology of drug rash with eosinophilia and systemic symptoms syndrome: a morphological and phenotypical study. Br J Dermatol.

[8] Chen C.B., Hung W.K., Wang C.W., Lee C.C., Hung S.I., Chung W.H. (2023). Advances in understanding of the pathogenesis and therapeutic implications of drug reaction with eosinophilia and systemic symptoms: an updated review. Front Med.

[9] Wei B.M., Fox L.P., Kaffenberger B.H. (2024). Drug-induced hypersensitivity syndrome/drug reaction with eosinophilia and systemic symptoms. Part I. Epidemiology, pathogenesis, clinicopathological features, and prognosis. J Am Acad Dermatol.

[10] Hama N., Abe R., Gibson A., Phillips E.J. (2022). Drug-induced hypersensitivity syndrome (DIHS)/drug reaction with eosinophilia and systemic symptoms (DRESS): clinical features and pathogenesis. J Allergy Clin Immunol Pract.

[11] Duong T.A., Valeyrie-Allanore L., Wolkenstein P., Chosidow O. (2017). Severe cutaneous adverse reactions to drugs. Lancet.

[12] Schroeder J.W., Napoli C., Caputo V., Bonoldi E., Rongioletti F. (2023). Unraveling the complexities of drug reaction with eosinophilia and systemic symptoms (DRESS): insights into clinical, laboratory, and histopathologic features of a case series from an Italian tertiary center. Clin Dermatol.

[13] Maverakis E., Ji-Xu A., Brüggen M.C. (2022). Targeting interleukin-5 with benralizumab: a novel treatment for drug rash with eosinophilia and systemic symptoms. Allergy.

[14] von Wachter C., Ameri M., Chimbetete T. (2025). Deciphering immune endotypes within drug reaction with eosinophilia and systemic symptoms (DRESS) syndrome. Allergy.

[15] Chiu T.M., Chen C.B., Lu C.W. (2025). CCR8/CCL1 and CXCR3/CXCL10 axis-mediated memory T-cell activation in patients with recalcitrant drug-induced hypersensitivity. Br J Dermatol.

[16] Chi KN, Agarwal N, Bjartell A, Chung BH, Gomes AJP de S, Given R (2019). Apalutamide for metastatic, castration-sensitive prostate cancer. N Engl J Med.

[17] Hsu YSO, Hsieh TS, Huang PW, Chu CY (2023). Drug reaction with eosinophilia and systemic symptoms with features resembling Stevens-Johnson syndrome/toxic epidermal necrolysis related to apalutamide. J Eur Acad Dermatol Venereol.

[18] Uemura H, Koroki Y, Iwaki Y, Imanaka K, Kambara T, Lopez-Gitlitz A (2020). Skin rash following Administration of Apalutamide in Japanese patients with Advanced Prostate Cancer: an integrated analysis of the phase 3 SPARTAN and TITAN studies and a phase 1 open-label study. BMC Urol.

[19] Tohi Y, Kato T, Fukuhara H, Kobayashi K, Ohira S, Ikeda K (2022). Real-world analysis of apalutamide-associated skin adverse events in Japanese patients with advanced prostate cancer: a multi-institutional study in the Chu-shikoku Japan Urological Consortium. Int J Clin Oncol.

[20] Akai N, Hashimoto T, Okuno S, Takai S, Satoh T (2025). Apalutamide-Induced Drug-Induced Hypersensitivity Syndrome/Drug Reaction With Eosinophilia and Systemic Symptoms Involving Reactivation of Human Herpesvirus-6 and Cytomegalovirus: A Case Report. J Dermatol.

[21] Gil-Lianes J, Amat-Samaranch V, Giavedoni P, Vilaseca A, Córdoba Sánchez J, Alamon-Reig F (2024 Sep). Acta Derm Venereol.

[22] Hallamies S, Auvinen R, Junkkari H, Kluger N (2024). Cutaneous adverse reactions associated to apalumide: two case reports of DRESS syndrome and maculopapular exanthema. Scand J Urol.

[23] Wang Q, Cao H, Zhang X, Wu H, Tang Z (2023). Case report: Apalutamide-induced severe lethal cutaneous adverse effects in China. Front Immunol.

[24] Flynn C.R., Liu S.C., Byrne B. (2023). Apalutamide-induced toxic epidermal necrolysis in a caucasian patient with metastatic castration-sensitive prostate cancer: a case report and review of the literature. Case Rep Oncol.

[25] Martin G, Lambert E, Wang GK (2023). Drug reaction with eosinophilia and systemic symptoms (DRESS) syndrome caused by apalutamide: a case presentation. Cureus.

[26] Ducharme O, Sanchez-Pena P, Pham-Ledard A, Beylot-Barry M, Milpied B (2022 Apr). The first case of drug reaction with eosinophilia and systemic symptoms (DRESS) syndrome caused by apalutamide, a novel oral androgen receptor antagonist. Contact Dermatitis.

[27] Sagawa N, Watanabe Y, Mizuno Y, Takahashi S, Watanabe T, Ikeda N (2020). A case of toxic epidermal necrolysis associated with apalutamide administration. J Cutan Immunol Allergy.

[28] Nguyen E, Yanes D, Imadojemu S, Kroshinsky D (2020). Evaluation of Cyclosporine for the Treatment of DRESS Syndrome. JAMA Dermatol.

[29] Wang S, Kang Y, He C, Jin H (2024). The systemic treatments for drug reaction with eosinophilia and systemic symptoms (DRESS) beyond corticosteroids. World Allergy Organ J.

